# Fraxin Alleviates Atherosclerosis by Inhibiting Oxidative Stress and Inflammatory Responses via the TLR4/PI3K/Akt Pathway

**DOI:** 10.3390/cimb47050308

**Published:** 2025-04-27

**Authors:** Yaru Wang, Bailing Wei, Mingyang Leng, Jiali He, Yicheng Zhao, Haohao Xia, Haibin Luo, Xue Bai

**Affiliations:** 1 Key Laboratory of Tropical Biological Resources of Ministry of Education, Hainan Engineering Research Center for Drug Screening and Evaluation, School of Pharmaceutical Sciences, Hainan University, Haikou 570228, China; 22211007000034@hainanu.edu.cn (Y.W.); 23110710000051@hainanu.edu.cn (B.W.); 24110710000035@hainanu.edu.cn (M.L.); 22220860020019@hainanu.edu.cn (J.H.); 23211007000025@hainanu.edu.cn (Y.Z.); 24221055000040@hainanu.edu.cn (H.X.); 2 School of Life and Health Science, Hainan University, Haikou 570228, China; 3 Song Li’ Academician Workstation, School of Pharmaceutical Sciences, Hainan University, Sanya 572000, China

**Keywords:** Fraxin, Atherosclerosis, TLR4, PI3K-Akt, reactive oxygen species

## Abstract

Fraxin is a bioactive compound derived from Cortex Fraxini. It is known for its diverse biological activities and numerous benefits, including anti-inflammatory, antioxidant, analgesic, antimicrobial, antiviral, and immunomodulatory effects. Despite growing interest in natural compounds for cardiovascular diseases Fraxin’s atheroprotective properties and molecular targets have not yet been fully elucidated. To address this gap, our research employed an integrated approach combining network pharmacology, molecular docking simulations, and in vitro biological validation to systematically unravel Fraxin’s therapeutic mechanisms against atherosclerosis (AS). The results showed that 84 potential targets for Fraxin against AS were predicted through public databases, and the key target TLR4 was identified by protein–protein interaction and molecular docking analysis. GO enrichment and KEGG pathway analysis revealed that these potential targets were significantly enriched in the PI3K-Akt and oxidative stress responses pathways. Subsequently conducted in vitro studies validated that Fraxin modulates the TLR4/PI3K/Akt signaling pathway to suppress reactive oxygen species generation and downregulate pro-inflammatory cytokines including *Il1b*, *Il6*, and *Tnf* thereby slowing atherosclerotic disease advancement. This investigation methodically delineates Fraxin’s therapeutic targets and underlying molecular mechanisms in AS management, establishing a scientific foundation for its potential translation into clinical practice.

## 1. Introduction

Atherosclerosis (AS), a chronic cardiovascular disorder, primarily manifests as lipid deposition at arterial branching sites, combined with excessive smooth muscle cell growth and the accumulation of extracellular matrix components [1,2,3]. According to the American Heart Association’s 2024 report on heart disease and stroke statistics, cardiovascular disease (CVD) stands as the primary contributor to illness and death across the globe. In 2021, CVD contributed to nearly 19.91 million global fatalities [4]. Due to the multifactorial nature and complex pathophysiology of AS, single-target therapies—such as statin-induced low-density lipoprotein (LDL) cholesterol reduction—often fail to fully arrest disease progression. While statins effectively lower LDL cholesterol, they may cause muscle-related adverse effects, including myalgia and rhabdomyolysis. Additionally, antiplatelet agents, like aspirin and P2Y12 receptor antagonists, increase bleeding risks [5,6]. These limitations underscore the need for novel therapeutic agents and targets.

Macrophages within the plaque microenvironment respond to stimuli such as cytokines and oxidized lipids, undergoing metabolic reprogramming and secreting pro-inflammatory or anti-inflammatory molecules [7]. Their uptake of oxidized low-density lipoprotein (ox-LDL) drives foam cell formation. These lipid-laden macrophages accumulate in the plaque core, elevating reactive oxygen species (ROS) production, promoting lipid peroxidation, and contributing to plaque necrosis and instability [8,9,10]. Evidence suggests that oxidative stress inhibition mitigates atherosclerotic lesion development [11]. The PI3K-Akt pathway, a central regulator of inflammation, autophagy, and lipid metabolism, plays a key role in AS pathogenesis [12,13,14].

For millennia, natural therapeutic agents have been employed extensively in the prevention and treatment of diverse pathological conditions, owing to their notable therapeutic efficacy and favorable safety profile, as demonstrated by extensive evidence [15]. In recent years, driven by societal progress and heightened interest in holistic health, natural therapeutic agents have increasingly been embraced across the globe. Notably, herbal medicine has gained significant recognition in numerous countries as a valuable complementary or alternative therapy option [16]. Coumarins have been recognized as preferred compounds among the phenolic compounds naturally found in various plant sources [17]. Fraxin, chemically known as 7-hydroxy-6-methoxycoumarin 8-glucoside, is a distinguished member of the coumarin class. It holds the position of the chief component in the traditional Chinese medicinal herb referred to as Cortex Fraxini. The multiple bioactivities of Fraxin, including anti-inflammatory, antioxidant, immune regulatory, anti-hyperuricemic, and diuretic properties, have been well-documented [18,19,20,21,22,23]. Nevertheless, the therapeutic potential of Fraxin in the prevention of atherosclerotic lesion formation has yet to be fully elucidated.

The current study was designed to explore the anti-atherosclerotic effects of Fraxin. Network pharmacology and molecular docking were utilized to determine potential molecular targets and biological pathways. In vitro experiments were then performed using RAW264.7 cell lines to confirm the molecular targets and physiological processes. The results of this research are intended to clarify the protective impacts and mechanisms of Fraxin in AS.

## 2. Materials and Methods

### 2.1. Collection of Potential Targets of Fraxin

By querying “Fraxin” in the PubChem database (https://pubchem.ncbi.nlm.nih.gov/) (accessed on 20 December 2024), we retrieved its canonical structure and SMILES representation. Using this information, potential molecular targets of Fraxin were predicted through the SEA Search Server (https://sea.bkslab.org/) (accessed on 20 December 2024), and SwissTargetPrediction (http://www.swisstargetprediction.ch/) (accessed on 20 December 2024), with the search restricted to “Homo sapiens”. The results from these databases were systematically curated through filtering, integration, and deduplication. Subsequently, all identified targets were standardized using the UniProt database (https://www.uniprot.org/) (accessed on 20 December 2024). This workflow culminated in the construction of a comprehensive and standardized target database for Fraxin, providing a basis for further pharmacological investigations.

### 2.2. Collection of Potential Targets of AS

Using GeneCards (https://www.genecards.org/) (accessed on 21 December 2024)and OMIM (https://omim.org/) (accessed on 21 December 2024) databases, we identified targets associated with “AS” through keyword search. To ensure a high correlation with AS, we implemented strict screening criteria and selected genes with high scores to establish a dedicated AS target gene pool. In addition, we also identified the common potential targets between aesculin targets and AS targets through Venn diagram analysis, and designated the overlapping part as the potential target of aesculin specifically affecting AS.

### 2.3. Construction of Protein–Protein Interaction Network

The overlapping targets were processed through the STRING database (https://string-db.org/) (accessed on 23 December 2024) to generate a protein–protein interaction network. A human-specific analysis was conducted with a medium confidence threshold (0.4) for interaction scores, while standard parameters were maintained. Cytoscape 3.9.1 software contributed network visualization, with central hub proteins identified through node connectivity metrics (degree values).

### 2.4. Gene Ontology (GO) and Kyoto Encyclopedia of Genes and Genomes (KEGG) Pathway Analyses

GO and KEGG pathway enrichment analyses were conducted using the DAVID database (https://david.ncifcrf.gov/) (accessed on 23 December 2024) to elucidate the biological functions of Fraxin’s potential targets in the context of AS. *p* < 0.05 was used as the screening value to obtain the corresponding data. GO evaluation systematically examined the functional annotations of candidate targets, categorized into three groups: biological processes, cellular localization, and molecular interactions. Concurrently, KEGG pathway analysis revealed principal signaling pathways linked to Fraxin’s anti-atherosclerotic activity. The results were visualized using online tools (https://www.bioinformatics.com.cn) (accessed on 23 December 2024).

### 2.5. Molecular Docking

The 2D structure of Fraxin was downloaded from PubChem. Energy minimization of the compound was conducted using ChemBio3D Ultra 14.0 software to derive its lowest-energy conformation. Three-dimensional structures of key target proteins were acquired from the PDB (https://www.rcsb.org/) (accessed on 29 December 2024). The AutoDock Tools (https://autodock.scripps.edu/) (accessed on 29 December 2024) was applied for preprocessing both the ligand and receptor files to enable binding simulations. Final docking outcomes were visualized and analyzed through PyMOL 3.1.3 to assess molecular interactions.

### 2.6. Cell Viability Assay

The murine macrophage lineage RAW264.7 was purchased from Wuhan Pricella Biotechnology Co., Ltd., (Wuhan, China). The cell culture medium consisted of DMEM (Gibco, Thermo Fisher Scientific, Waltham, MA, USA) with 10% FBS and 1% penicillin-streptomycin (100 μg/mL penicillin, 100 μg/mL streptomycin). Cells were cultured at 37 °C with 5% CO_2_ and treated with 75 μg/mL ox-LDL (Unionbiol, Beijing, China) for stimulation. Metabolic activity was quantified using a CCK-8 assay per manufacturer’s instructions (Solarbio, Beijing, China). Cells were plated in 96-well format at 2 × 10^4^ viable cells/well and allowed to stabilize for 24 h under standard conditions. Subsequently, the cells were pretreated with Fraxin (PRF24102221; Biopurify Phytochemicals Ltd., Chengdu, China) at concentrations of 5, 10, 20, 40, 80, 160, 320, and 640 μmol/L for 24 h. Following pretreatment, the complete medium was exchanged for serum-free medium containing CCK-8 reagent for subsequent assays. The CCK-8 reagent was incubated for 1 h at 37 °C before measuring optical density measurements were obtained at 450 nm.

### 2.7. Oil-Red O Staining

RAW264.7 macrophages were carefully plated in 24-well culture dishes containing 8 × 10^4^ cells in each well and subsequently treated with Fraxin (5, 10, and 20 μmol/L) in combination with 75 μg/mL ox-LDL for 48 h. After the incubation period, cellular fixation was performed using 4% paraformaldehyde for 20 min. Subsequently, the specimens were subjected to two washes with PBS and treated with 60% isopropanol. Following a 30-min incubation with oil red O, cellular samples were subjected to 60% isopropanol washes to eliminate unbound dye. Nuclear staining was performed using hematoxylin for 2 min and subsequently counterstained with 0.1% NaOH for 15 s. We observed and imaged intracellular lipid droplets stained red under an inverted microscope (Leica, Berlin, Germany).

### 2.8. Quantification of Total Cholesterol (TC) Content

Following 24-h co-treated with Fraxin (5, 10, and 20 μmol/L) and 75 μg/mL ox-LDL, cells were pelleted by centrifugation and homogenized in absolute ethanol using freeze-thaw cycles alternating between liquid nitrogen and 37 °C to ensure complete cell lysis. TC content was then quantified using a commercial TC assay kit (Elabscience, Wuhan, China) according to the manufacturer’s protocol, with absorbance measured at 510 nm using a microplate reader (Agilent, Santa Clara, CA, USA) for concentration determination.

### 2.9. Determination of Glutathione (GSH) and Malondialdehyde (MDA) Content

Cells were co-treated with Fraxin (5, 10, and 20 μmol/L) and 75 μg/mL ox-LDL for 24 h. Subsequently, GSH (Solarbio, Beijing, China, BC1175) and MDA (Nanjing Jiancheng, Nanjing, China, A003-1-2) content were quantified using commercial assay kits according to the manufacturer’s protocols. Absorbance was measured at 412 nm (GSH) and 532 nm (MDA) using a microplate reader (Agilent, Santa Clara, CA, USA).

### 2.10. RNA Extraction and qRT–PCR Analysis

RAW264.7 macrophages were plated in 12-well culture dishes (3 × 10^5^ cells/well) and subsequently co-treated with Fraxin (5, 10, 20 μmol/L) and ox-LDL (75 μg/mL) for 24 h. Cellular RNA was isolated from cultured RAW264.7 macrophages using TRIzol reagent (Invitrogen, Carlsbad, CA, USA) with subsequent quantification and quality assessment conducted on a NanoDrop 2000 spectrophotometer (Thermo Fisher Scientific, Waltham, MA, USA). We reverse-transcribed 1000 ng total RNA into cDNA following the PrimeScript RT protocol (TaKaRa, Tokyo, Japan, RR047A), then conducted real-time PCR analysis using TB Green Premix (TaKaRa, Tokyo, Japan, RR820A) on a Roche LightCycler 480 platform (Roche, Basel, Switzerland). Quantitative analysis of gene expression was conducted using the 2^−ΔΔCt^ calculation algorithm. Corresponding primer sequences are tabulated in Table 1.

### 2.11. Western Blot Assay

Protein extracts obtained from RAW264.7 cells using inhibitor-supplemented RIPA buffer (Beyotime Biotechnology, Haimen, China) were quantified (BCA assay, Thermo Fisher Scientific, Waltham, MA, USA), electrophoresed (25 μg), and transferred to PVDF membranes (0.22 μm). To prevent nonspecific antibody binding, the immunoblot membrane was saturated with 5% fetal bovine serum under standard temperature conditions (RT, 20–25 °C) for 60 min. Next, the primary antibody was incubated at 4 °C overnight using the following specific antibodies: phospho-PI3K (1:1000, AF5905, Beyotime Biotechnology, Haimen, China), PI3K (1:1000, AF7742, Beyotime Biotechnology, Haimen, China), phospho-Akt (1:2000, #4060S, Cell Signaling Technology, Danvers, MA, USA), Akt (1:1000, #9272S, Cell Signaling Technology, Danvers, MA, USA), TLR4 (1:1000, #CSB-PA001434, CUSABIO, Wuhan, China), and β-actin (1:3000, #AF7018, Affinity, Springfield, NJ, USA). We performed secondary antibody incubation with HRP-conjugated monoclonal antibody (1:1000, #A0208, Beyotime Biotechnology, Haimen, China) maintained at room temperature (approximately 25 °C) for 1 h. Finally, membranes visualized by using the ImageQuant 800 (Cytiva, Cardiff, UK).

### 2.12. ROS Detection Assay

The effect of Fraxin on ROS production was assessed using a laser confocal micro-scope. Prior to experimental interventions, murine RAW264.7 macrophages were seeded in 24-well culture plates at a density of 8 × 10^4^ cells per well. We exposed cells to Fraxin (5, 10, 20 μmol/L) in combination with 75 μg/mL ox-LDL for 24 h. After aspiration of the complete medium, adherent cells were rinsed twice with serum-depleted medium using gentle pipetting. Subsequently, we added DCFH-DA probe at 1:1000 dilution in serum-depleted medium following the washing steps. We maintained cells at 37 °C for 30 min, then counterstained nuclei with Hoechst 33342 (10 min incubation). Final visualization was conducted via confocal fluorescence microscopy (OLYMPUS, Tokyo, Japan).

### 2.13. Data Processing and Statistical Analyses

Quantitative data are presented as arithmetic mean ± SEM (*n* ≥ 3). Using GraphPad Prism version 9.5, we performed ordinary one-way ANOVA for multiple comparisons, considering *p* < 0.05 statistically significant.

## 3. Results

### 3.1. Network Pharmacology Prediction

#### 3.1.1. Identification of Targets of Fraxin Against AS

**Figure 1:**
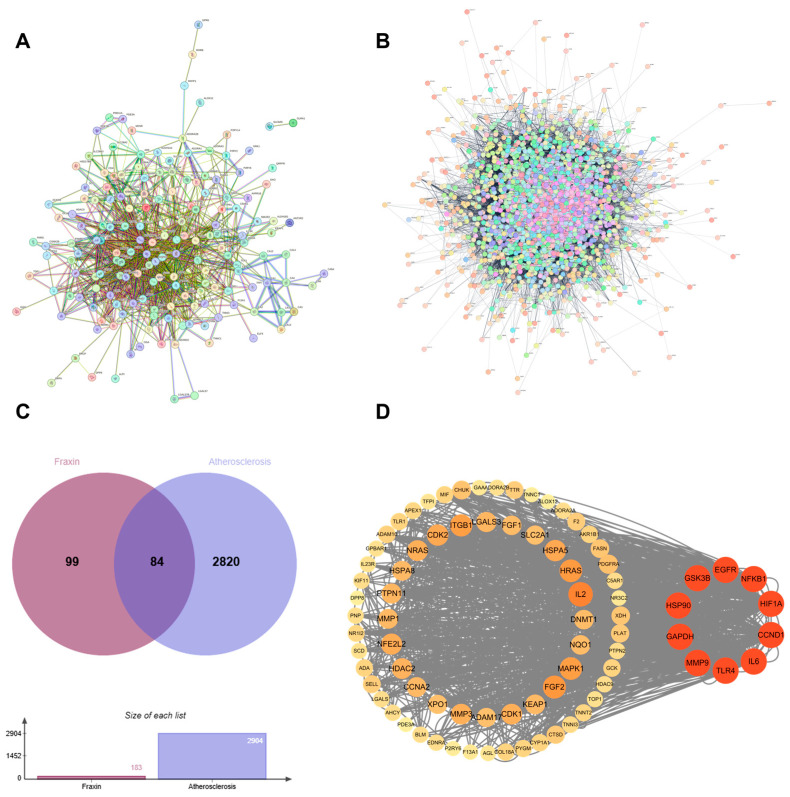
Investigating the effect of Fraxin on AS based on network pharmacology. (**A**) 183 Fraxin-associated targets were collected. (**B**) 2904 AS-related targets were collected. (**C**) Venn diagram of the targets of Fraxin and AS. (**D**) The PPI network of potential targets. A larger area indicates larger nodes, a red color indicates higher association, lighter color less association, and the core target is the target in the right circle.

A multi-database computational target mining strategy was implemented for Fraxin, combining SEA Search Server with Swiss Target Prediction databases. After screening and removing duplicates, 183 non-redundant targets underwent protein–protein interaction (PPI) network topology analysis via STRING (Figure 1A). Additionally, targets related to AS were predicted using the GeneCards and OMIM databases. Following further screening and the removal of duplicates, a total of 2904 potential targets were obtained (Figure 1B). The two networks were combined to form an overlapping network with Fraxin and AS as core components. A cross-database analysis revealed 84 overlapping biomolecules between Fraxin-associated proteinsand atherosclerotic pathomechanisms, establishing these molecular entities as prioritized candidates for subsequent anti-atherosclerosis validation studies (Figure 1C).

#### 3.1.2. Protein–Protein Interaction Network

The PPI network of the 84 convergence targets was performed using STRING followed by network visualization in Cytoscape 3.9.1. The resultant interactome map comprised 84 nodes and 619 edges, demonstrating a densely interconnected architecture. Based on the degree values, we selected the top ten genes (TLR4, IL6, GAPDH, HIF1A, EGFR, HSP90, MMP9, NFKB1, CCND1, GSK3B), which are considered crucial in Fraxin’s treatment of AS and potential key targets (Figure 1D).

#### 3.1.3. GO and KEGG Pathway Enrichment Analysis

**Figure 2:**
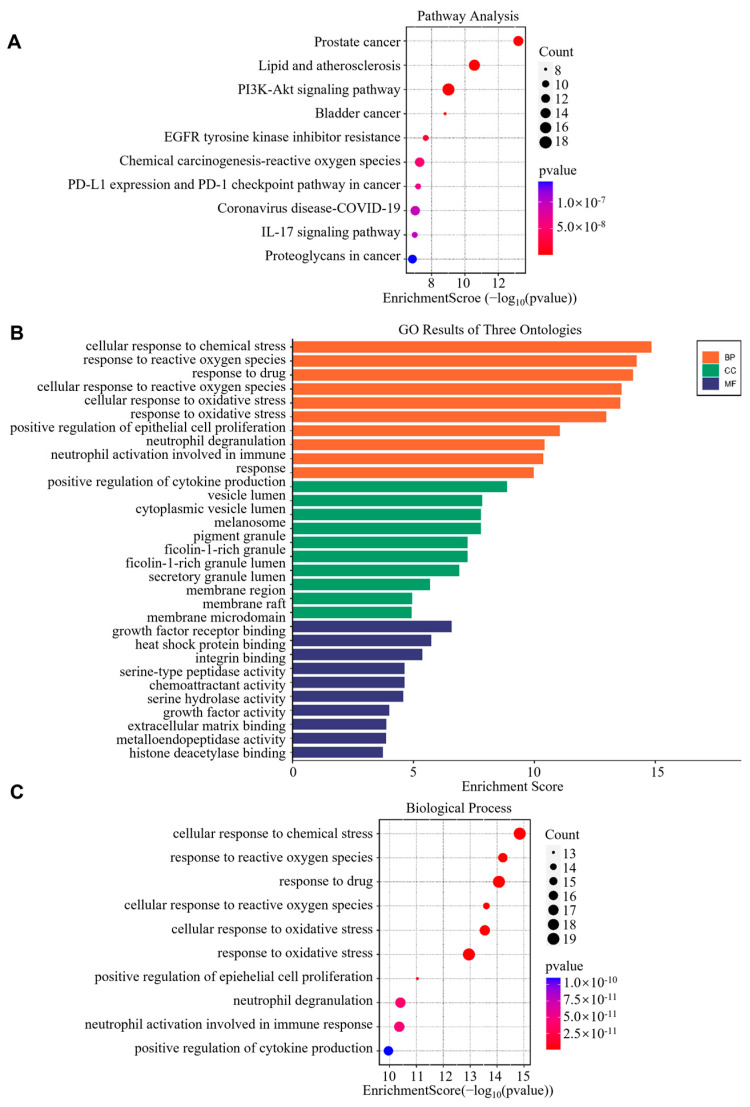
KEGG and GO enrichment analysis of potential targets. (**A**) KEGG enrichment analysis of 84 common targets of Fraxin and AS. (**B**) GO analysis of 84 common targets of Fraxin and AS. (**C**) Biological processes in the GO analysis of 84 common targets of Fraxin and AS.

To delineate Fraxin’s anti-atherosclerotic mechanisms, functional enrichment analyses (GO/KEGG) of the 84 overlapping targets was performed. The KEGG enrichment results revealed that these 84 intersecting targets were significantly enriched in pathways such as the PI3K-Akt signaling pathway, EGFR tyrosine kinase inhibitor resistance, lipid and AS, and the IL-17 signaling pathway. Notably, the PI3K-Akt signaling pathway exhibited the highest number of enriched genes and may serve as a key pathway through which Fraxin exerts its effects (Figure 2A). Secondly, the GO analysis results indicated that, although Fraxin functions in multiple cellular regions and interacts with other proteins to influence various important biological processes, it is highly enriched in pathways related to oxidative stress. These pathways include the response to reactive oxygen species, cellular response to reactive oxygen species, cellular response to oxidative stress, and response to oxidative stress (Figure 2B,C). This suggests that Fraxin may improve AS by alleviating oxidative stress. Therefore, we hypothesize that Fraxin may enhance AS outcomes by regulating oxidative stress through the PI3K-Akt signaling pathway.

#### 3.1.4. Verification of Results by Molecular Docking

**Figure 3:**
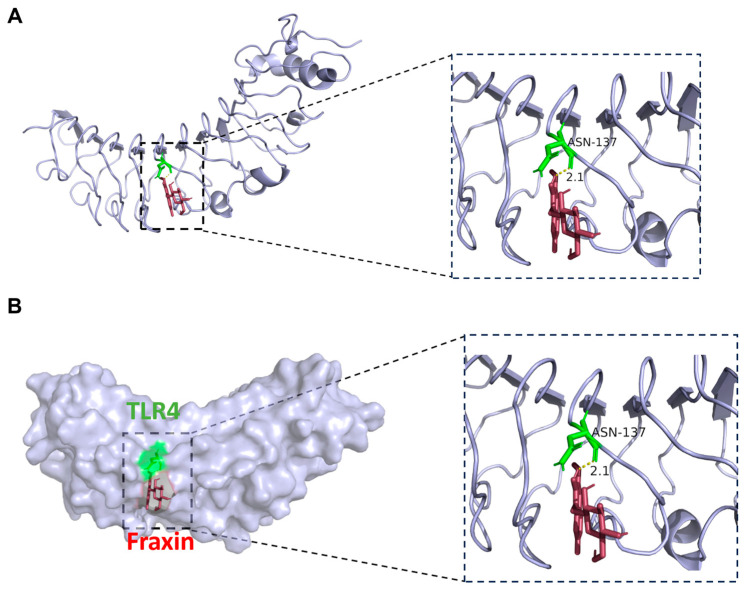
Molecular docking results. (**A**,**B**) Molecular model of Fraxin binding to the predicted TLR4 target. Red represents Fraxin, while green indicates the binding sites between TLR4 and Fraxin.

TLR4, a canonical pattern-recognition receptor (PRR) governing innate immune responses, functions as a primary regulatory node for PI3K-Akt cascade activation through MyD88-dependent phosphorylation events. Therefore, we conducted molecular docking validation between Fraxin and TLR4 and found that Fraxin forms a hydrogen bond with ASN-137(2.1 A) in TLR4, implicating this ligand-receptor axis as the predominant mechanistic route for PI3K-Akt pathway regulation in atherosclerotic contexts (Figure 3A,B).

### 3.2. In Vitro Experimental Verification

#### 3.2.1. Fraxin Inhibits Lipid Accumulation and Reduces Cholesterol Content in Macrophages

**Figure 4:**
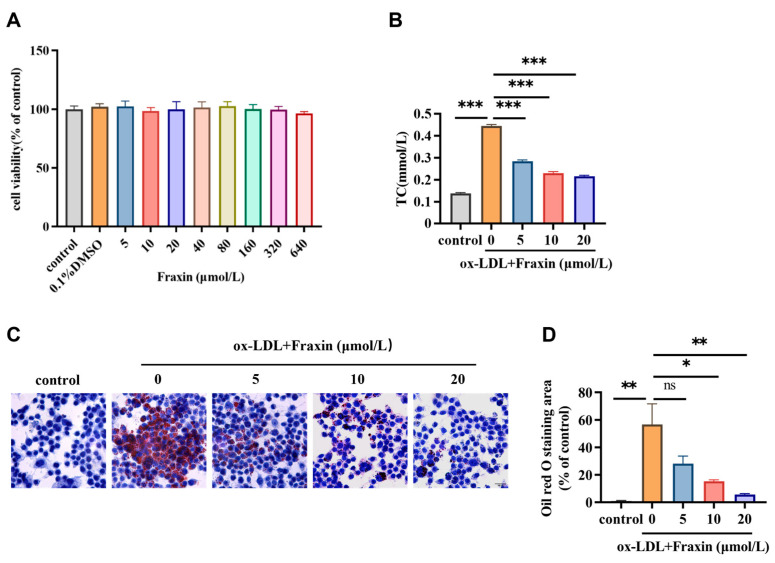
Changes in cholesterol and lipid contents with Fraxin effects. (**A**) The cytotoxic effects of Fraxin on RAW264.7 cells were evaluated after 24 h treatment, with viability determined by CCK-8 absorbance measurem. (**B**) Dose-dependent effects of Fraxin on ox-LDL (75 μg/mL)-induced intracellular cholesterol accumulation in RAW264.7 cells. (**C**) Lipid deposition detected by Oil Red O staining. Cells were examined by light microscope (20× magnification). Scale bar indicates 100 μm. (**D**) The percentage of lipid-stained area by Oil Red O in each group relative to control group cells. All values are means ± SEM (*n* = 3 in each group). *ns* = not significant (*p* ≥ 0.05), * *p* < 0.05, ** *p* < 0.01, *** *p* < 0.001.

In vitro study first investigated the cytotoxicity of Fraxin on RAW264.7 macrophage cells using the CCK-8 kit (Figure 4A). Fraxin-treated cells maintained comparable viability to untreated controls across tested concentration gradients, with no dose-dependent cytotoxic effects observed. Therefore, we concluded that Fraxin exhibited no cytotoxicity at concentrations up to 640 μmol/L. Foam cells, characterized by the accumulation of cholesterol esters, contribute to AS progresses. Ox-LDL stimulation increases intracellular cholesterol levels, leading to macrophage death and subsequent necrosis of surrounding cells, which is associated with plaque formation and disruption. The results obtained through chemical analysis indicated that Fraxin significantly reduced cholesterol content attributed to ox-LDL (Figure 4B). The detection of foam cell formation is conventionally performed using Oil Red O (ORO) staining to identify lipid-laden macrophages. We found that Fraxin significantly inhibited lipid accumulation (Figure 4C), and quantification of lipid-stained areas further demonstrated that Fraxin suppressed lipid deposition in a dose-dependent manner (Figure 4D).

#### 3.2.2. Fraxin Inhibits TLR4/PI3K/Akt Pathway and Suppresses Macrophage Inflammatory

**Figure 5:**
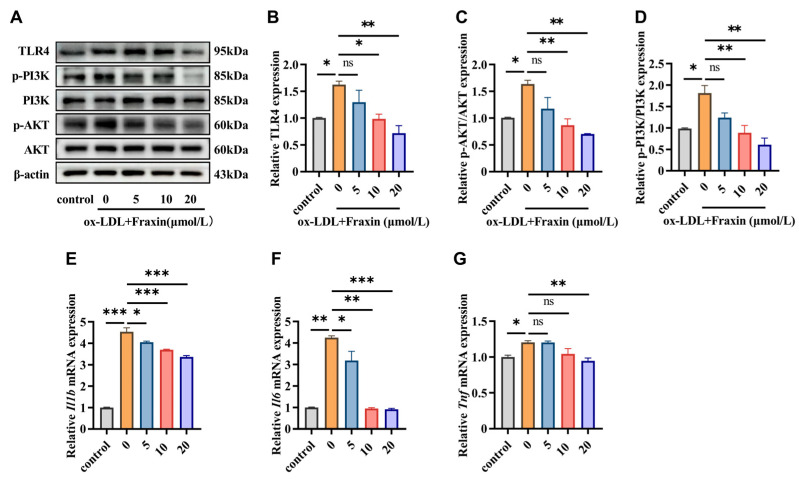
Effect of TLR4/PI3K/Akt pathway and inflammatory factors for RNA level with Fraxin. (**A**) TLR4, p-PI3K, PI3K, p-Akt, Akt protein were detected by Western blot. (**B**–**D**) The quantification of TLR4, p-PI3K/PI3K and p-Akt/Akt relative expression levels. (**E**–**G**) Fraxin-mediated modulation of pro-inflammatory cytokines (*Il1b*, *Il6*, *Tnf*) in ox-LDL-stimulated RAW264.7 macrophages. All values are means ± SEM (*n* = 3 in each group). *ns* = not significant (*p* ≥ 0.05), * *p* < 0.05, ** *p* < 0.01, *** *p* < 0.001.

TLR4-mediated PI3K/Akt pathway activation plays a pivotal role in driving inflammatory responses and promoting foam cell generation during AS development [24]. Suppressing PI3K activity or blocking Akt phosphorylation effectively downregulates NLRP3 inflammasome activation, subsequently lowering interleukin-1β (IL-1β) release and impairing macrophage capacity to internalize ox-LDL, which collectively contributes to attenuated foam cell accumulation and reduced atherosclerotic progression [25]. Our experimental data demonstrated that ox-LDL exposure (75 μg/mL, 24 h) induced significant upregulation of TLR4 protein expression and enhanced PI3K/Akt phosphorylation in RAW264.7 macrophages. Notably, Fraxin effectively attenuated these pathological signaling alterations, as evidenced by Western blot (Figure 5A–D). Tumor Necrosis Factor-α (TNF-α) and IL-1β are critical in driving the inflammatory processes underlying AS. Their roles vary depending on the stage of disease, with TNF-α being more prominent in early atherogenesis and IL-1β in advanced plaque inflammation [26,27]. Additionally, Interleukin-6 (IL-6) can enhance the production of other pro-inflammatory cytokines, like TNF-α and IL-1β, further amplifying the inflammatory response [28]. Our study showed the transcription level of 3 key inflammation factors, *Il1b*, *Il6*, and *Tnf* with ox-LDL stimulating different times. The results indicated that Fraxin at 20 μmol/L significantly reversed a sharp increase in *Il1b*, *Il6*, *Tnf* mRNA contents. (Figure 5E–G).

#### 3.2.3. Fraxin Significantly Reverses the Redox Steady State Disrupted by ox-LDL

**Figure 6:**
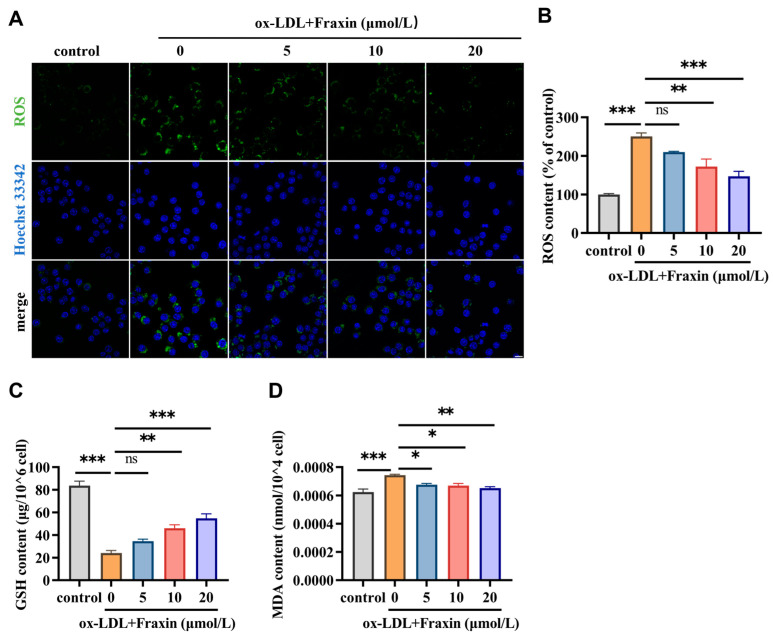
Effect of Fraxin on oxidative stress of ox-LDL-induced RAW264.7. (**A**) Oxidative stress was assessed through fluorescent probe-based imaging analysis. Scale bar indicates 10 μm. (**B**) ROS content under different Fraxin concentrations. (**C**) GSH contet under different Fraxin concentrations. (**D**) MDA content under different Fraxin concentrations. All values are means ± SEM (*n* = 3 in each group). *ns* = not significant (*p* ≥ 0.05), * *p* < 0.05, ** *p* < 0.01, *** *p* < 0.001.

Oxidative stress in macrophages plays a crucial role in driving inflammation, foam cell formation, and plaque instability [29]. ox-LDL is a potent inducer of oxidative stress in macrophages [9]. The oxidation process of ox-LDL involves free radical mediated lipid peroxidation, leading to damage to the lipid bilayer and inflammatory response, glutathione plays a core role in regulating the redox state within cells [30]. In this study, the ox-LDL triggered ROS accumulation and GSH loss in the macrophages was markedly recovered in the presence of Fraxin (Figure 6A–C). Moreover, MDA is an unstable aldehyde compound with high reactivity. Under acidic conditions, the interaction between MDA and thiobarbituric acid (TBA) yields a characteristic red-colored TBA–MDA adduct, which serves as a standard assay for quantifying lipid peroxidation levels. Further results showed that Fraxin significantly attenuated lipid peroxidation due to ox-LDL in RAW264.7 cells (Figure 6D).

#### 3.2.4. Overexpression of TLR4 Abrogated the Effects of Fraxin

**Figure 7:**
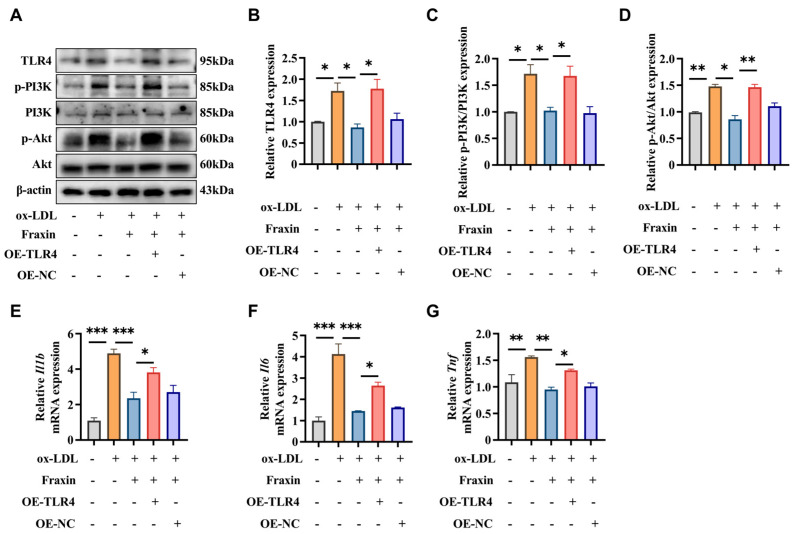
The effect of Fraxin was reversed by TLR4. (**A**) Western blot analysis was performed to examine protein expression levels of TLR4, p-PI3K, PI3K, p-Akt, and Akt in Fraxin-treated cells with TLR4 overexpression (OE-TLR4) and corresponding negative controls (OE-NC). (**B**–**D**) The quantification of TLR4, p-PI3K/PI3K and p-Akt/Akt relative expression levels. (**E**–**G**) TLR4 overexpression modulates the secretion of pro-inflammatory cytokines (*Il1b*, *Il6*, *Tnf*). All values are means ± SEM (*n* = 3 in each group). * *p* < 0.05, ** *p* < 0.01, *** *p* < 0.001.

To validate the TLR4/PI3K/Akt pathway in Fraxin-mediated attenuation of atherosclerotic progression, gain-of-function studies were performed by transfecting RAW264.7 macrophages with TLR4-overexpressing plasmids. Intriguingly, TLR4 overexpression (OE-TLR4) abrogated Fraxin’s suppressive effects on PI3K/Akt, as evidenced by increased p-PI3K/PI3K and p-Akt/Akt ratios (Figure 7A–D). The results strongly corroborated our hypothesis of pathway dependency. Further characterization demonstrated that TLR4 overexpression markedly elevated proinflammatory cytokine (*Il1b*, *Il6*, and *Tnf*) levels (Figure 7E–G). Collectively, these experiments establish that Fraxin exerts its anti-atherosclerotic effects through modulation of the TLR4/PI3K/Akt signaling cascade.

## 4. Discussion

**Figure 8:**
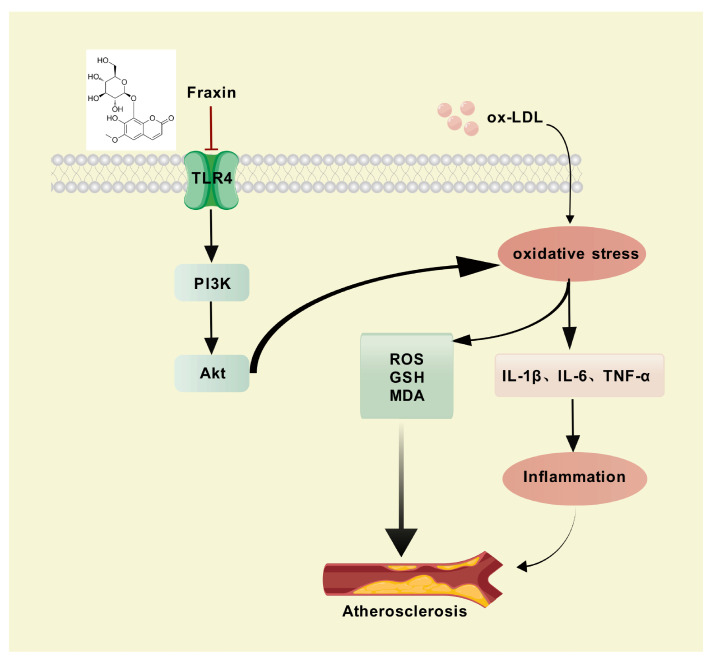
Proposed mechanisms underlying Fraxin anti-atherosclerosis by inhibiting TLR4/PI3K/Akt pathway and oxidative stress. Created with biogdp.com.

This investigation synergized network pharmacology with functional cell-based analyses to systematically decode Fraxin’s anti-atherosclerotic mechanisms, establishing a multi-omics framework that bridges computational prediction and biological validation. Relative research on the function of Fraxin gave potential information to inhibition of AS using related disease and drug target databases. Subsequently, we reorganized the identical genes of drug targets and disease targets and displayed this information through Vene plots. Through functional annotation of core targets and pathway enrichment analysis, we established a mechanism framework elucidating Fraxin’s therapeutic effects on AS progression. Protein–protein interaction network analysis of 84 candidate targets identified 10 hub targets, including TLR4, IL6, GAPDH, and MMP-9. Subsequent multi-omics integration revealed these targets’ involvement in critical pathological processes, such as lipid and AS, particularly through modulation of the PI3K/Akt axis. GO results indicated that Fraxin joined response of reactive oxygen species as well as contribute to oxidative stress suppression, consistent with biological process analysis. These results suggest that Fraxin exerts therapeutic effects against AS by modulating key targets and related signaling pathways (Figure 8).

The formation of foam cells, marked by pathological cholesterol/lipid deposition, critically drives necrotic core expansion in atherosclerotic plaques [31]. Our research integrated network pharmacology and in vitro validation and revealed Fraxin’s dual inhibitory effects on cholesterol biosynthesis and lipid accumulation. Moreover, AS is a chronic inflammatory response associated with various inflammatory factors [32]. IL-6, a potent proinflammatory cytokine implicated in atherosclerotic pathogenesis, stimulates the synthesis of downstream inflammatory mediators, including TNF-α and IL-1β, thereby exacerbating the pro-inflammatory cascade [28]. In this study, Fraxin significantly reduced the mRNA expression of *Il6*, *Il1b*, and *Tnf* (Figure 5E–G). We therefore proceeded to investigate the specific mechanism through which Fraxin exerts its effects. Through in-depth analysis of our predicted results, we observed that TLR4 appears to play a critical role in this process. To further validate the interaction between Fraxin and TLR4, molecular docking was performed, revealing their capacity to form a stable binding complex (Figure 3). We hypothesize that Fraxin is likely to mediate its effects via TLR4 and its downstream signaling cascade.

TLR4 is a quintessential pattern-recognition receptor in the innate immune response and is crucial for triggering inflammation in AS [33,34]. Its expression is markedly upregulated in macrophages within atherosclerotic lesions [35]. Furthermore, TLR4 activation prompts the release of various cytokines and chemokines that are key to recruiting and proliferating inflammatory cells. Consequently, this sets off an inflammatory cascade that facilitates the buildup of foam cells in the intima [36]. Our findings similarly demonstrated significant upregulation of TLR4 expression in ox-LDL-treated macrophages, while Fraxin administration induced a dose-dependent attenuation of TLR4 protein levels (Figure 5A,B).

Pathway analysis revealed that Fraxin exerts its anti-atherosclerotic effects primarily through the PI3K/Akt signaling pathway and oxidative stress-related pathways (Figure 2). Previous studies have demonstrated that Fraxin exhibits potent antioxidant capacity by stimulating antioxidant enzymes, downregulating NF-κB and NLRP3 inflammasome activation, and activating the Nrf2/ARE signaling pathway, thereby mitigating oxidative stress-associated damage in various organs [37]. In AS, oxidative stress is a key driver of inflammatory responses, activating the inflammatory cascade and recruiting macrophages to the vascular wall [38,39,40]. Additionally, oxidative stress causes abnormal modification of LDL, leading to impaired vasodilation, promoting plaque formation, and accelerating the progression of AS [41]. Studies have shown that inhibiting oxidative stress in macrophages can effectively suppress plaque formation, exerting a positive effect on AS [42]. As a critical regulator of inflammatory responses, the PI3K/Akt signaling cascade has been extensively documented to drive AS progression when dysregulated [43]. Notably, TLR4-dependent PI3K/Akt signaling is closely associated with the oxidative stress and inflammatory responses [44]. Therefore, we further investigated the effects of Fraxin on the PI3K/Akt pathway and oxidative stress. Experimental outcomes demonstrated that Fraxin attenuated PI3K and Akt phosphorylation in a concentration-dependent manner, concurrently reducing intracellular ROS and MDA accumulation while restoring GSH to baseline levels (Figure 4 and Figure 5). To more precisely confirm that Fraxin exerts its effects via TLR4-mediated the PI3K/Akt pathway, we performed TLR4 overexpression. After TLR4 overexpression, the PI3K/Akt pathway inhibited by Fraxin was activated, the expression of inflammatory factors was restored, and the therapeutic effect of Fraxin was nullified (Figure 7). These results more definitively indicate that Fraxin exerts its effects through the TLR4/PI3K/Akt signaling pathway.

Collectively, our findings highlight Fraxin’s therapeutic potential in mitigating AS. Fraxin ameliorates AS by suppressing the TLR4-mediated PI3K-Akt signaling pathway, thereby mitigating inflammatory and oxidative stress responses. These findings suggest that Fraxin holds promise as a clinical candidate for the treatment of AS.

## Figures and Tables

**Table 1:** Primer sequences.

Gene Name	Forward Primer	Reverse Primer
*Il1b*	gaaatgccaccttttgacagtg	tggatgctctcatcaggacag
*Il6*	ctgcaagagacttccatccag	agtggtatagacaggtctgttgg
*Tnf*	caggcggtgcctatgtctc	cgatcaccccgaagttcagtag
*Gapdh*	aggtcggtgtgaacggatttg	tgtagaccatgtagttgaggtca

## Data Availability

The datasets used and/or analyzed during the current study are available from the corresponding author on reasonable request.
